# Supplementary material for: Functionally specialized human CD4+ T-cell subsets express physicochemically distinct TCRs
Source: eLife. 2020 Dec 8;9:e57063. doi: 10.7554/eLife.57063 (PMC7773335; doi:10.7554/eLife.57063)
Supplement: Transparent reporting form [file elife-57063-transrepform.docx]

***eLife’s* transparent reporting form**

We encourage authors to provide detailed information *within their submission* to facilitate the interpretation and replication of experiments. Authors can upload supporting documentation to indicate the use of appropriate reporting guidelines for health-related research (see [EQUATOR Network](http://www.equator-network.org/%20)), life science research (see the [BioSharing Information Resource](https://biosharing.org/)), or the [ARRIVE guidelines](http://www.plosbiology.org/article/info:doi/10.1371/journal.pbio.1000412) for reporting work involving animal research. Where applicable, authors should refer to any relevant reporting standards documents in this form.

If you have any questions, please consult our Journal Policies and/or contact us: [editorial@elifesciences.org](mailto:editorial@elifesciences.org).

**Sample-size estimation**

- You should state whether an appropriate sample size was computed when the study was being designed
- You should state the statistical method of sample size computation and any required assumptions
- If no explicit power analysis was used, you should describe how you decided what sample (replicate) size (number) to use

Please outline where this information can be found within the submission (e.g., sections or figure legends), or explain why this information doesn’t apply to your submission:

This work is focused on deep sequencing of individual TCR alpha and beta repertoires for CD4 T cells, rather than measuring a single statistic for large groups of donors. We indicate in the manuscript that the observed subset-specific characteristics were reproducible across donors, but do not claim that it will be the same across population. The achieved depth of TCR profiling (thousands of clonotypes per sample) was limited by amount of sorted CD4 subset T cells, but was sufficient for repertoire features analysis and investigation of CD4 T cells plasticity and repertoire publicity.

**Replicates**

- You should report how often each experiment was performed
- You should include a definition of biological versus technical replication
- The data obtained should be provided and sufficient information should be provided to indicate the number of independent biological and/or technical replicates
- If you encountered any outliers, you should describe how these were handled
- Criteria for exclusion/inclusion of data should be clearly stated
- High-throughput sequence data should be uploaded before submission, with a private link for reviewers provided (these are available from both GEO and ArrayExpress)

Please outline where this information can be found within the submission (e.g., sections or figure legends), or explain why this information doesn’t apply to your submission:

Samples were collected and sequenced without replicates.

Sequencing data were deposited in GEO under accession code GSE158848.

**Statistical reporting**

- Statistical analysis methods should be described and justified
- Raw data should be presented in figures whenever informative to do so (typically when N per group is less than 10)
- For each experiment, you should identify the statistical tests used, exact values of N, definitions of center, methods of multiple test correction, and dispersion and precision measures (e.g., mean, median, SD, SEM, confidence intervals; and, for the major substantive results, a measure of effect size (e.g., Pearson's r, Cohen's d)
- Report exact p-values wherever possible alongside the summary statistics and 95% confidence intervals. These should be reported for all key questions and not only when the p-value is less than 0.05.

Please outline where this information can be found within the submission (e.g., sections or figure legends), or explain why this information doesn’t apply to your submission:

Figure 2. One-way ANOVA followed by the two-sample Welch t-test with Bonferroni correction for each group versus the mean. CD4+ T cell subsets (n = 8) from each healthy donor (n = 5).

Figure 3. One-way ANOVA followed by the two-sample Welch t-test with Bonferroni correction for each group versus the mean. CD4+ T cell subsets (n = 8) from each healthy donor (n = 5).

Figure 4A. One-way ANOVA followed by the two-sample Welch t-test with Bonferroni correction for each group versus the mean. CD4+ T cell subsets (n = 8) from each healthy donor (n = 5).

Figure 6A. One-way ANOVA followed by the two-sample Welch t-test with Bonferroni correction for each group versus the mean. CD4+ T cell subsets (n = 3) from each healthy donor (n = 12).

Figure 6B. One-way ANOVA followed by the two-sample Welch t-test with Bonferroni correction for each group versus the mean. CD4+ T cell subsets (n = 5) from each healthy donor (n = 4).

(For large datasets, or papers with a very large number of statistical tests, you may upload a single table file with tests, Ns, etc., with reference to sections in the manuscript.)

**Group allocation**

- Indicate how samples were allocated into experimental groups (in the case of clinical studies, please specify allocation to treatment method); if randomization was used, please also state if restricted randomization was applied
- Indicate if masking was used during group allocation, data collection and/or data analysis

Please outline where this information can be found within the submission (e.g., sections or figure legends), or explain why this information doesn’t apply to your submission:

CD4+ T cell samples were allocated into functional subset groups.

**Additional data files (“source data”)**

- We encourage you to upload relevant additional data files, such as numerical data that are represented as a graph in a figure, or as a summary table
- Where provided, these should be in the most useful format, and they can be uploaded as “Source data” files linked to a main figure or table
- Include model definition files including the full list of parameters used
- Include code used for data analysis (e.g., R, MatLab)
- Avoid stating that data files are “available upon request”

Please indicate the figures or tables for which source data files have been provided:

Obtained TCR repertoires and metadata are available at <https://figshare.com/s/2145b1b16c6854445af7> and <https://figshare.com/s/84ec5f412356afb0536d>
